# A retrospective case series of the treatment of spontaneous quadriceps tendon rupture in patients with uremia and secondary hyperparathyroidism

**DOI:** 10.3389/fsurg.2023.961188

**Published:** 2023-02-23

**Authors:** Shuang Wu, Huihui Wang, Yanlin Zhu, Weili Fu

**Affiliations:** ^1^ Department of Orthopedics, West China Hospital, Sichuan University, Chengdu, China; ^2^ Department of Orthopedics, Provincial Orthopaedics Hospital, Chengdu, China

**Keywords:** Spontaneous quadriceps tendon rupture, Secondary hyperparathyroidism, uremia quadriceps tendon rupture, uremia, secondary hyperparathyroidism, parathyroidectomy, ligament reconstruction

## Abstract

**Background:**

Spontaneous quadriceps tendon rupture (QTR) is a rare complication of uremia. Secondary hyperparathyroidism (SHPT) is considered the leading cause of QTR in uremia patients. QTR in patients with uremia and SHPT are treated with active surgical repair along with the treatment of SHPT using medication or parathyroidectomy (PTX). The impact of PTX for SHPT on tendon healing remains unclear. The purpose of this study was to introduce surgical procedures for QTR and to determine the functional recovery of the repaired quadriceps tendon (QT) after PTX.

**Methods:**

Between Jan 2014 and Dec 2018, eight uremia patients underwent PTX after a ruptured QT was repaired by figure-of-eight trans-osseous sutures with an overlapping tightening suture technique. Biochemical indices were measured before and one year after PTX to evaluate the control of SHPT. The changes in bone mineral density (BMD) were determined by comparing x-ray images at pre-PTX and during follow-up. The assessment of the functional recovery of the repaired QT was conducted at the last follow-up using multiple functional parameters.

**Results:**

Eight patients (fourteen tendons) were retrospectively evaluated at an average follow-up of 3.46 ± 1.37 years after PTX. ALP and iPTH levels one year after PTX were significantly lower than at pre-PTX (*P* = 0.017, *P *< 0.001, respectively). Although there was no statistical differences compared to pre-PTX, serum phosphorus levels decreased and returned to normal one year after PTX (*P* = 0.101). BMD significantly increased at the last follow-up compared to pre-PTX. The average Lysholm score was 73.5 ± 11.07 and the average Tegner activity score was 2.63 ± 1.06. The active knee ROM after repair averaged an extension of 2.85 ± 3.78° to a flexion angle of 113.21 ± 10.12°. The quadriceps muscle strength was grade IV and the mean Insall-Salvati index was 0.93 ± 0.10 in all of the knees with tendon ruptures. All patients were able to walk without external help.

**Conclusions:**

Figure-of-eight trans-osseous sutures with an overlapping tightening suture technique is an economical and effective treatment for spontaneous QTR in patients with uremia and SHPT. PTX may promote tendon-bone healing in patients with uremia and SHPT.

## Introduction

A spontaneous tendon rupture may be defined as a rupture that occurs during movement activities that should not usually damage the involved musculo-tendinous units (1). In the absence of a significant history of trauma, spontaneous quadriceps tendon rupture (QTR) is often misdiagnosed or delayed in patients with chronic renal failure (CRF) where the tendons have become weakened, predisposing them to ruptures (2). According to previous reports, spontaneous QTR is a rare complication in hemodialysis patients with uremia. Since the first description by Steiner and Plamer (3), only over 100 cases have been documented. Complete QTR must be treated surgically to regain full function recovery, however, the underlying cause should be quickly diagnosed to allow the prompt commencement of treatment.

In uremic patients, the mechanisms of QTR is controversial and include secondary hyperparathyroidism (SHPT), corticosteroid treatment, metabolic acidosis, post-dialysis amyloidosis, malnutrition, and the accumulation of uremic toxins (4, 5). Of these factors, SHPT is considered the leading cause of tendon rupture in uremia patients (6–8).

The management of patients with SHPT is predominantly medical. Despite advances in medical therapy for SHPT, parathyroidectomy (PTX) remains the definitive therapy for refractory SHPT (9). However, there are currently only a few reports on the comprehensive treatment of spontaneous QTR in patients with uremia and SHPT. Also, the influence of PTX for SHPT after tendon repair on tendon healing remains unclear.

In this study, we report a case series of 8 patients with uremia and SHPT who suffered from spontaneous QTR. All patients had undergone quadriceps tendon (QT) repair at our Institution followed by PTX for SHPT. We aimed to determine the functional recovery of the repaired QT after PTX.

## Methods

### Participants

This retrospective study was approved by our Institutional review board and patients were recruited under informed consent. Between Jan 2014 and Dec 2018, ten uremia patients with SHPT suffered from complete QRTs at our Institution. Of these patients, two patients were lost at follow-up and so eight patients were included in this study. None of the patients had a history of steroid and fluoroquinolone administration.

Table 1 summarizes the patient characteristics of those included in this study. The median age of the patients was 38 ± 9.93 years. Males were more often affected than females (7:1). The average BMI was 21.95 ± 2.11 kg/m^2,^ and the average period of hemodialysis was 5.38 ± 2.07 years. The mean time from the injury to QT repair was 14.13 ± 14.35 weeks. Six patients presented with simultaneous bilateral QTRs and one patient showed a unilateral QTR. One patient presented a QT and contralateral patellar tendon rupture but had a four-month interval between the lesions. The mechanism of injury in both bilateral and unilateral QTR was most commonly a slip or fall on a flat surface (four patients). This was followed by falls on stairs (two patients) and injuries resulting from falling from a bicycle (one patient). Also one patient was injured by sudden exertion whilst trying to stand up from squatting (Table 1).

**Table 1:** Summary of the patient characteristics.

Patient	Age	Gender	BMI	Rupture	Mechanism	Dialysis Time	Rupture to surgery interval
	(years)	(M/F)	(kg/m^2^)	side	of injury	(years)	(weeks)
1	32	M	21.1	Bilateral	d	4	21
2	28	M	21.6	Bilateral	a	3	40
3	24	F	18.8	Right	c	4	1
4	50	M	22.1	Bilateral	a	7	5
5	48	M	23.8	Bilateral	b	9	2
6	33	M	25.9	Bilateral	b	5	26
7	46	M	21.1	Bilateral	a	7	17
8	43	M	21.2	Left	a	4	1

a = slip and fall on a flat surface; b = a fall down the stairs; c = a fall from a bicycle; d = sudden exertion whilst trying to stand up from squatting.

Laboratory findings showed increased intact parathyroid hormone (iPTH), serum phosphorus, and alkaline phosphatase (ALP) in all patients (Table 2). x-ray imaging revealed severe and generalized osteoporosis and showed patella baja. Calcifications were observed in the ruptured ends of the tendon in all patients. MRI revealed the quadricep tendons presented an avulsion-like rupture at their osteotendinous junction.

**Table 2:** Summary of the laboratory findings for the patients recruited to the study.

Patient	Ca	*P*	AG	ALP	iPTH	UREA	CREA	25-OH-VD
	(mmol/L)	(mmol/L)	(mmol/L)	(IU/L)	(pmol/L)	(mmol/L)	(umol/L)	(nmol/L)
	[2.1–2.7]	[0.67–1.04]	[12.0–20.0]	[51–160]	[1.60–6.90]	[3.30–8.22]	[53.0–140.0]	[47.7–144]
1	2.3	2.94	30.5	827	370.1	21.8	935	54.24
2	2.43	2.38	23.2	307	175.3	17.5	906	72.33
3	2.39	2.51	28.6	1088	146	30.2	868	63.1
4	2.2	2.06	21.7	358	183.5	27.7	811	57.86
5	2.06	2.76	27.8	362	243.7	23.6	1042	41.62
6	2.03	1.72	17.9	401	190.8	13.2	551	32.61
7	2.07	2.48	26.7	374	218.4	22.79	763	35.72
8	2.25	1.87	19.9	1794	333.2	13.9	644	56.95

Ca, calcium; P, phosphorus; AG, anion gap; ALP, alkaline phosphatase; iPTH, intact parathyroid hormone; CREA, creatinine; 25-OH-VD, 25- hydroxyvitamin D.

### Surgical techniques

**Figure 1:**
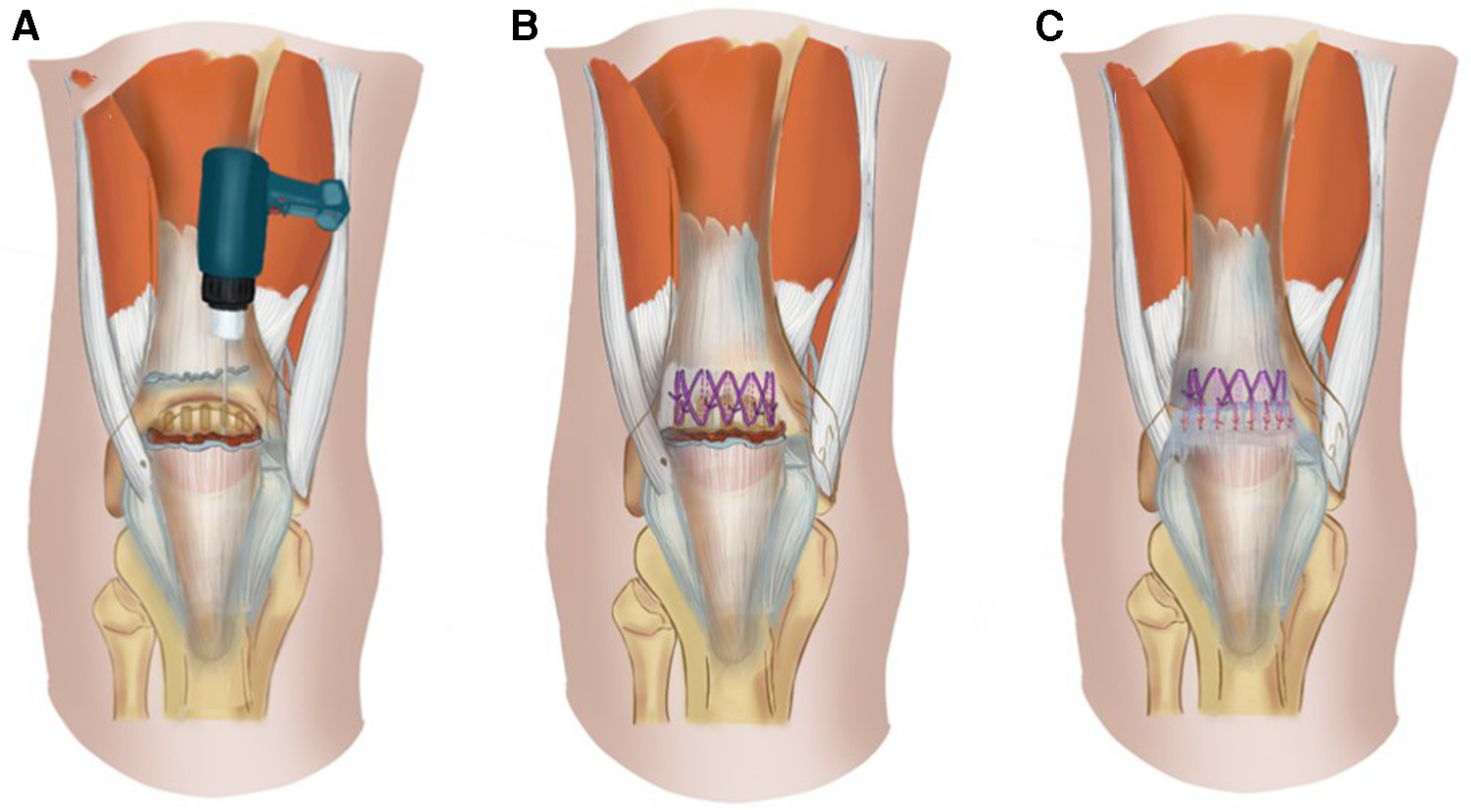
Diagram of the QT repair surgery. (**A**). Five parallel longitudinal trans-osseous tunnels with diameters of 2.0 mm were made in the upper pole of the patella using a drill. (**B**). A figure-of-eight suture was performed through each of the adjacent bone tunnels and simple trans-osseous sutures were achieved through the bone tunnels on both sides. (**C**). Tears in the retinaculum and deep fascia on the surface of the patella were repaired using an overlapping tightening suture with absorbable suture material.

All ruptured QTs were repaired using figure-of-eight trans-osseous sutures with an overlapping tightening suture technique *via* an anterior median longitudinal approach. A longitudinal incision of 8 cm is made from the proximal part of the QT tear site to the upper pole of the patella. Non-viable tendon extremities were debrided and any grossly degenerative tissue was excised. The end of the tendon was freshened and the surface of the upper pole of the patella roughened using a spherical burr. Five parallel longitudinal trans-osseous tunnels with diameters of 2.0 mm were made in the upper pole of the patella using a drill (Figure 1A). A non-absorbable number 5 Ethibond suture material was used for trans-osseous sutures. A figure-of-eight suture was performed through each of the adjacent bone tunnels and simple trans-osseous sutures placed on the bone tunnels on both sides (Figure 1B). The suture was tied with the knee in full extension. Tears in the retinaculum and deep fascia on the surface of the patella were repaired using an overlapping tightening suture with absorbable No. 1-0 sutures (Ethicon) (Figure 1C). No additional reinforcing systems were used.

### Post-operative rehabilitation

The knee was locked in full extension with a cylinder cast or a range-of-motion brace for a minimum of 6 weeks post-operatively. Patients were allowed partial weight-bearing with their cylinder cast or brace locked in extension immediately after surgery(10). Electrical stimulation was performed routinely. At seven weeks after the operation, patients were permitted to undertake full weight-bearing activities and progressive range of motion (ROM) exercise was maintained (10 days at 0°, 30°, 60°, and 90°) aiming for full flexion by six weeks.

### PTX for SHPT

After consulting the multiple disciplinary teams (MDT) including the departments of nephrology, endocrinology and thyroid surgery, all patients underwent PTX (11) after repairing the ruptured tendon.

### Clinical and functional assessment

The serum levels of calcium, phosphorus, ALP and iPTH before and one year after PTX were measured to evaluate the control of SHPT. Changes in bone mineral density (BMD) was determined by comparing x-ray images at pre-PTX and the final follow-up.

We attempted to have all patients return to assess the functional outcomes of the knee. Patients who returned fulfilled a Lysholm score and Tegner activity score. The results of QT repair were evaluated with particular attention to the knee ROMs measured using a goniometer (0°corresponding to full extension). The quadriceps muscle strength was reported based on the Kendall scale (12). In cases where the patient agreed, isokinetic muscle strength (IMS) tests were performed (IsoMed-2000; D&R Ferstl, Hemau, Germany). Also, the ability to walk without crutches and the occurrence of new ruptures after the procedure were recorded.

Anteroposterior (AP) and lateral radiographs with the knee flexed at 30° were taken. The patella height was measured according to Insall and Salvati index (the ratio of the longest patellar diagonal length to tendon length with the knee in 30° of flexion. A value of ≤0.8 indicated that the patella baja was present.

### Statistics analysis

Continuous variables were expressed as the mean ± standard deviation, and categorical variables were expressed as frequencies. Differences in the serum levels of calcium, phosphorus, ALP, and iPTH at pre-PTX and one year after PTX were compared using a paired-samples T-test. Probability values of *P* less than 0.05 were considered significant. All statistical analyses were performed using SPSS 18.0.

## Results

Eight patients (fourteen tendons) were included in this study. The mean time from the injury to QT repair was 14.13 ± 14.35 weeks. The average time from QT repair to PTX was 10.33 ± 1.53 weeks. The average follow-up after PTX was 3.46 ± 1.37 years. Intra-operative observations confirmed all QTs presented an avulsion-like rupture at the osteotendinous junction.

Table 3 shows SHPT was well controlled in all patients one year after PTX. ALP and iPTH levels one year after PTX were significantly lower than that the levels at pre-PTX (*P* < 0.05). Serum phosphorus levels decreased and returned to normal one year after PTX. No statistical differences in serum phosphorus levels were detected one year after PTX compared to pre-PTX levels. Serum calcium levels were not significantly different at pre-PTX and one year after PTX. BMD was shown to significantly increase at the last follow-up compare to pre-PTX measurements.

**Table 3:** Changes in the biochemical indices of patients before and one year after PTX.

Variable	Pre PTX	Post PTX 1 year	*P*-value
Ca (2.1-2.7 mmol/L)	2.43 ± 0.25	2.31 ± 0.31	0.408
*P* (0.67–1.04 mmol/L)	2.15 ± 0.55	1.68 ± 0.52	0.101
ALP (51-160IU/L)	732.83 ± 634.21	117.43 ± 112.64	0.017*
iPTH (1.60–6.90 pmol/L)	232.47 ± 86.35	4.65 ± 4.21	<0.001*

Ca, calcium; P, phosphorus; ALP, alkaline phosphatase; iPTH, intact parathyroid hormone; 25-OH-VD, 25-hydroxyvitamin D; NA, not available; PTX, parathyroidectomy.

* *P *< 0.05.

The outcome scores at the last follow-up are summarized in Table 4. The patients were satisfied with the treatment results. The average Lysholm score was 73.5 ± 11.07 and the average Tegner activity score was 2.63 ± 1.06. Active knee ROM after repair averaged an extension of 2.85 ± 3.78° to flexion of 113.21 ± 10.12°. Quadriceps muscle strength in all knees was grade IV according to the Kendall scale. Only one patient performed IMS tests (Table 5) and the results are shown in the subsequent case report. All patients were able to walk without external help. Post-operative x-rays showed good positioning of the patella. The mean Insall-Salvati index was 0.93 ± 0.10 in all the knees with tendon ruptures (Table 4).

**Table 4:** Functional evaluation at the last follow-up.

Patient	ROM (°)	Insall-Salvati ratio			The quadriceps muscle strength		Tegner activity score	Lysholm score	Complications
	R	L	R	L	R	L			
1	0–120	5–115	0.93	1.01	IV	IV	3	75	-
2	0–125	0–115	0.81	0.84	IV	IV	4	86	skin breakdown
3	0–130	0–140	O.91	NA	IV	V	4	94	-
4	10–105	0–115	0.86	0.82	IV	IV	2	65	-
5	0–95	5–100	1.07	0.96	IV	IV	1	63	delayed healing
6	0–120	10–115	0.92	0.87	IV	IV	3	70	-
7	5–110	0–120	1.02	0.89	IV	IV	2	65	-
8	0–130	5–100	NA	1.13	V	IV	2	70	-

ROM, Range of motion; NA, not available.

**Table 5:** Changes in biochemical indices and BMD before and after PTX and the IMS test results at one year after PTX.

Variable	Pre PTX	Post PTX 2 week	Post PTX 1 year	Variable	R	
Ca (2.1–2.7 mmol/L)	2.47	1.78	2.12	IMS testing result		
P (0.67–1.04 mmol/L)	1.99	0.64	0.72	FTW (J)	359	485
ALP (51–160IU/L)	2005	3157	365	ETW (J)	599	695
iPTH (1.60–6.90 pmol/L)	352.6	4.79	7.82	FPT (N·m)	33	48
26-OH-VD (47.7–144 nmol/L)	39.1	NA	174.6	EPT (N·m)	71	80
BMD value (g/cm^2^)/ T score				The relative FPT(N·m/kg)	0.66	0.96
L1-4 lumbar spine	0.689/−3.5	NA	1.156/0.4	The relative EPT (N·m/kg)	1.42	1.6
Left femoral neck	0.691/−2.0	NA	1.064/1.1			
Left total hip	0.726/−1.9	NA	1.008/0.3			

Ca, calcium; P, phosphorus; ALP, alkaline phosphatase; iPTH, intact parathyroid hormone; 25-OH-VD, 25-hydroxyvitamin D; NA, not available; PTX, preparathyroidectomy. T score > −1.0 equals normal BMD, −2.5 < T value < −1.0 denotes osteopenia/low bone mass, and T value < −2.5 indicates osteoporosis. IMS, Isokinetic Muscle Strength; FTW, total work of flexion; ETW, total work of extension; FPT, Peak Torque of flexion; EPT, The peak torque of extension; relative FPT:FPT/BW; relative EPT:EPT/BW.

### Complications

Complications associated with quadriceps tendon repair included delayed wound healing in one patient and skin breakdown from casting in another patient. Both of these complications were resolved. No deep infections and no new ruptures occurred during the follow-up periods.

### Illustrative case (case 3)

A 24-year-old woman who had been receiving hemodialysis for four years for CRF suffered acute pain and swelling in her right knee after a trivial fall from a bicycle at low speed. The patient could not stand or walk after the fall and could not actively extend the right knee. A palpable depression was present in the suprapatellar region. The medical treatment of the patient consisted of amlodipine (10 mg/day), Bisoprolol (10 mg/day), Irbesartan (300 mg/day), Calcitriol (0.5 µg/day) and calcium supplements (500 mg/day). The patient was lean with a body mass index of 18.8 kg/m^2^.

**Figure 2:**
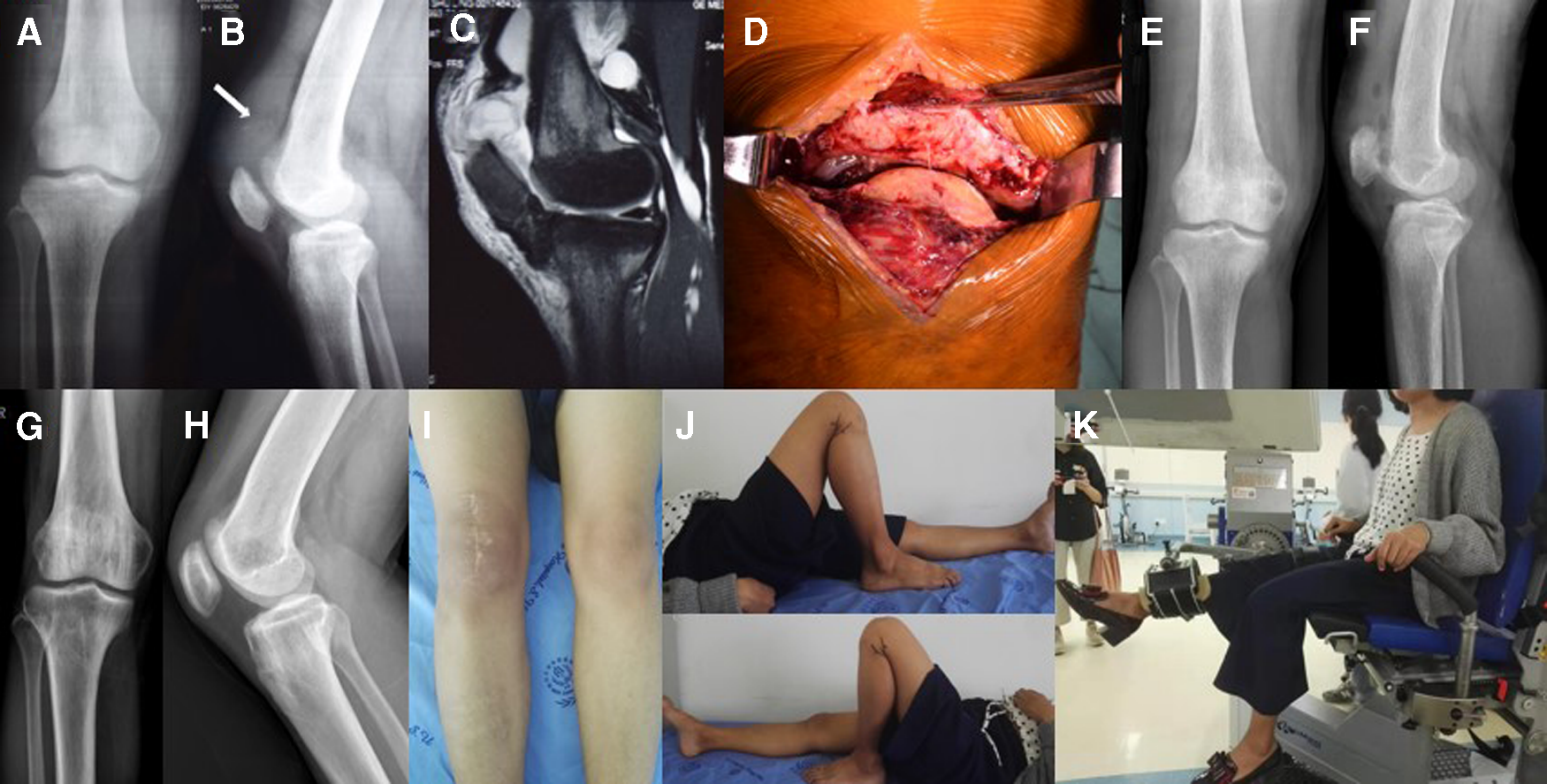
(**A**) x-ray of the right knee taken at the initial consultation showing severe osteoporosis. (**B**) Lateral radiograph showing the patella baja and calcification in the ruptured end of the QT (arrow). (**C**) Lateral T2-weighted MRI of the right knee showing a complete disruption of the QT at the patellar insertion sites. (**D**) The QT peel off from the upper pole of the patella. (**E,F**) Radiography after QT repair showing the regular positioning of the patella. (**G,H**) Radiography one year after PTX showed BMD had significantly increase compared to pre-PTX levels. (**I,J**) One year after PTX, the ROM of the right knee was 0–130° and in the left knee was 0–140°. (**K**) The patient undergoing IMS tests.

Preoperative examination showed that serum levels of phosphorus, anion clearance, ALP, iPTH, urea, and creatinine were higher than normal. Levels of 25-OH-VD were lower than normal and blood calcium was normal suggesting SHPT (Table 2, Patient 3). A lateral radiograph revealed an abnormal patella baja and calcification at the end of the ruptured tendon. The patient was also seen to have severe osteoporosis (Figures 2A,B). MRI revealed a complete disruption of the quadriceps tendon at the patellar insertion sites (Figure 2C).

The patient was diagnosed with a unilateral spontaneous QTR and SHPT with secondary osteoporosis. Surgical intervention was recommended. The MDT consultation recommended PTX after QT repair.

One week after the injury, the patient underwent surgery during which an avulsion-like rupture at the osteotendinous junction was found (Figure 2D). The tendon was repaired using a trans-osseous suture with a figure-of-eight stitch *via* an anterior knee approach as described above.

Postoperative radiography showed that the patella height had returned to normal (Figures 2E,F). After a six-week immobilization period, the patient was allowed full weight-bearing and continuous passive-active movements to exercise the extensor mechanism and improve flexion range. Three months after surgery, the patient regained 130° flexions without an extension lag. Walking was possible without external help and the monopodial standing position in flexion was stable.

**Figure 3:**
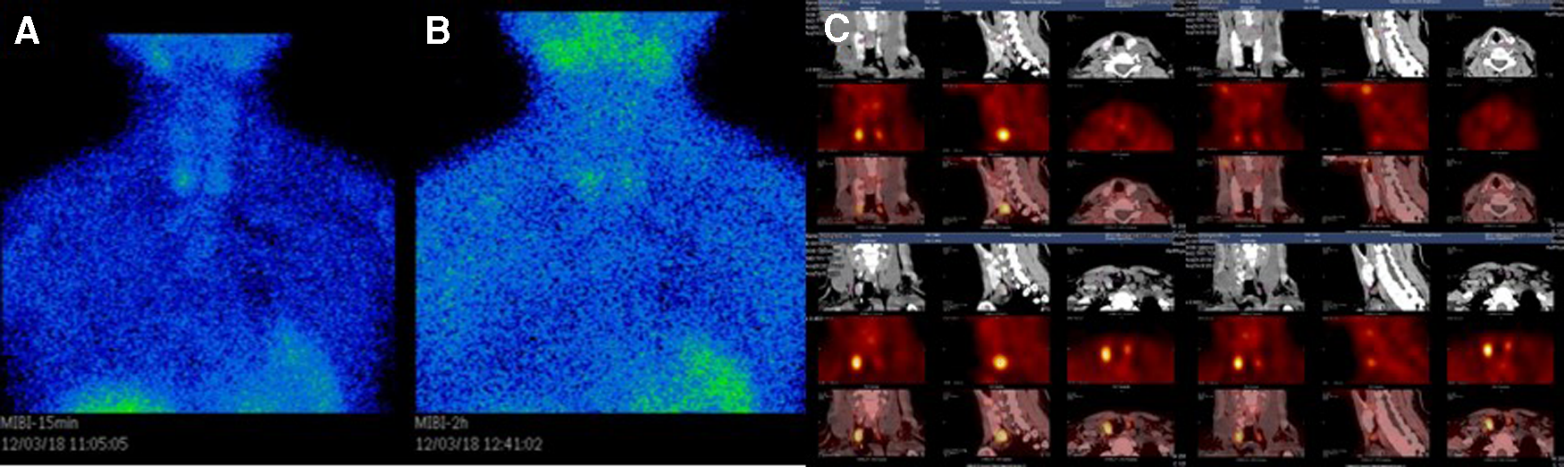
(**A**) 99mTc-MIBI dual-phase planar imaging. The early phase (15 min) planar images showed MIBI uptake in the inferior pole of the right thyroid lobe. (**B**) The delayed phase (2 h) planar images showed MIBI uptake in the inferior and upper pole of the bilateral thyroid lobes. (**C**) 99mTc-MIBI SPECT/CT imaging showing small soft-tissue masses with MIBI uptake in the back of the inferior Poles of both bilateral thyroid lobes.

Twelve weeks after the QT repair, the patient found that her height had decreased significantly from normal 162 to 151 cm, and she felt pain in her heel when walking. Examinations showed an abnormal increase of 352.60 pg/ml in iPTH. 99mTc-MIBI dual-phase planar imaging showed MIBI uptake in the inferior pole of the right thyroid lobe in the early phase (15 min) (Figure 3A), and the inferior and upper pole of the bilateral thyroid lobes in the delayed phase (2 h) (Figure 3B). 99mTc-MIBI SPECT/CT imaging showed small soft-tissue masses with MIBI uptake in the back of the inferior poles of both bilateral thyroid lobes (Figure 3C). DXA BMD examination showed that the patient had severe osteoporosis (Table 5). The patient was diagnosed with tertiary hyperparathyroidism (THPT) (13) and shrinking man syndrome (14). She underwent total PTX and autografting of the brachioradialis muscle in the right forearm of the upper left parathyroid gland. Postoperative histopathology confirmed that the mass was hyperplastic parathyroid tissue. After the operation, the patient presented with hungry bone syndrome (HBS) (15).

One year after PTX, the patient was back to normal life and the iPTH had returned to normal. ALP had decreased significantly (Table 5). The right quadriceps muscle strength based on the Kendall scale was IV/V. IMS tests (Figure 2K) showed the strength of extension and flexion on the operated side had decreased compared to the normal side (Table 5). The height of the patient increased by 4 cm without obvious bone pain and movement dysfunction. x-ray imaging of the knee (Figures 2G,H) and BMD examination revealed a significant increase in BMD compared to pre-PTX levels (Table 5).

## Discussion

In this retrospective study, we investigated the injury characteristics, surgical procedures and outcomes of spontaneous QTR in patients with uremia and SHPT. Also, we investigated PTX for the treatment of SHPT and determined its effects on tendon-bone healing. All QTs presented an avulsion-like rupture at the osteotendinous junction and were repaired using figure-of-eight trans-osseous sutures with an overlapping tightening suture technique. All patients underwent PTX after QT repair and parathyroid function was well controlled. The knee joint function recovered satisfactorily at the last follow-up. After repair of the QT in patients with uremia and SHPT, PTX may promote tendon-bone healing.

The mechanisms of QTR in patients with uremia remain controversial. They include malnutrition, *β*-2- amyloidosis, chronic acidosis, vitamin D deficiency, accumulation of uremia toxins, and SHPT (5, 16–19). In uremia patients undergoing maintenance hemodialysis, chronic acidosis can cause tendon degeneration (20). The accumulation of uremic toxins affects collagen structure and metabolism (21) as well as the soft tissue deposition of amyloid (22) that can weaken tendons. These pathological changes may lead to tendon rupture in the tendon or at the musculotendinous junction, rather than at the tendon-bone junction (23). Subperiosteal bone resorption and dystrophic calcifications due to SHPT may lead to tendon rupture with minor trauma.

The high level of parathyroid hormone (PTH) stimulates osteoclast activity which induces subtendinous bone reabsorption. This results in structural entheseal modifications that lead to the avulsion of the tendinous insertion. Finally, repeated minor avulsion fractures result in total tendon ruptures with minor trauma (23). SHPT also leads to dystrophic calcification which reduces the elasticity of the tendon and causes fragility of the bone tendon junction (24).

In this study, all patients presented with SHPT as determined by the laboratory and based on radiologic findings. The ruptured site was just at the tendon attachment to the bone, which was smooth and completely free from tendon tissue. All quadricep tendons presented a calcification in the ruptured end of the tendon. They were consistent with the findings reported by Shiota et al. (23) who studied five long-term dialysis patients with seven spontaneous ruptures of major tendons and found that the ruptured site was at the osteotendinous junction. These data suggest that the most likely cause of tendon rupture in hemodialysis patients is SHPT.

When the QTR occurs in patients with uremia and SHPT, in addition to the active surgical repair of the ruptured tendon, the treatment of the main risk factors leading to tendon rupture should also be carried out as soon as possible. Consistent with most reports (3, 25) for the surgical treatment of QTR, we used non-absorbable no. 5 Ethibond suture to attach the ruptured tendons onto the patella which prevented late failure due to hardware fatigue and avoided the need to later remove hardware. The two most common techniques used for rupture closure to the patellar insertion are trans-osseous sutures (gold standard) or suture anchors, both of which have yielded excellent outcomes (26–28).

In our study, considering bone resorption, osteoporosis, and health economics, we opted for trans-osseous sutures. In most case reports, trans-osseous sutures are commonly used in combination with Bunnel or Krakow sutures and a suture-bone tunnel technique (23, 25). In the current cases, the proximal tendon was sutured using a figure-of-eight suture to improve tissue grip and avoid tight sutures that affect the blood supply of the tendon and can impact tendon-bone healing. Furthermore, the torn retinaculum and deep fascia on the surface of the patella was sutured to the QT using an overlapping tightening suture technique to strengthen the ruptured tendon. All of our patients achieved excellent results and no re-rupture occurred at the last follow-up. Figure-of-eight trans-osseous sutures with an overlapping tightening suture technique was an economical and highly effective treatment for this avulsion-like rupture.

Some authors hypothesize that a missed diagnosis may lead to delayed repair, which could be problematic due to the significant retraction of the quadriceps. Treatment would therefore require more complex surgical techniques to fix the consequent defect and restore the extensor system of the knee (29). In contrast, the current study found that patients with delayed surgery could have their quadriceps tendon directly stitched together, without the no need for lengthening plasty and tendon grafting to compensate for retraction. These findings were consistent with the previous results from Malta et al. (12). We found that there was no apparent defect in the length of the quadriceps tendon for this avulsion-like rupture. The uremia patients were complicated with muscle atrophy and decreased muscle tone and so it was easy to pull the ruptured QT back to the upper pole of the patella. Komaba et al^.^ (9) also reported that many patients with CRF exhibit signs of wasting and muscle atrophy. Also, some studies have shown that SHPT may be involved in the pathogenesis of wasting and muscle weakness (30–33).

During treatment, uremia patients should be identified as soon as possible to ensure that patients complicated with SHPT are given timely treatment to prevent and control SHPT (34). The strict control of SHPT is crucial to avoid tendon rupture or re-rupture (23). In our study, one patient presented QTR four months after contralateral patellar tendon rupture due to delayed diagnosis and treatment of SHPT. If SHPT had been diagnosed and treated at the time of the initial tendon rupture in this patient, the second episode of the tendon rupture could likely have been prevented. In addition to tendon ruptures, SHPT can also lead to severe complications such as pathological fractures, shrinking man syndrome and vascular calcification (34, 35). The longer SHPT patients are exposed to high serum iPTH, the more likely they are to have serious complications (36). Moreover, patients with long-standing SHPT can experience parathyroid hyperplasia and develop THPT (36). In addition, studies have shown that electrical stimulation during the postoperative rehabilitation phase can delay muscle disuse atrophy after knee ligament injury to some extent (37).

Currently, there are no clear criteria to aid in the treatment decisions for SHPT in patients with uremia and spontaneous QTR. Various treatment options are available including drug treatment and surgery. Although pharmacologic treatments for SHPT are generally successful in reducing PTH levels, results vary by drug class and are frequently limited by several factors including medication adherence, adverse events associated with medications and high pill burden (38, 39). Despite advances in the treatment of SHPT, PTX remains the definitive therapy for refractory SHPT (9).

PTX is more effective than drug therapy in controlling the symptoms of patients, the associated biochemical indices (40) and significantly improves patient quality of life (41). Furthermore, circulating PTH levels drastically decreases after PTX followed by progressive reductions in serum calcium and phosphorus levels. This condition is known as a hungry bone syndrome and is characterized by massive deposition of calcium and phosphate in the bone resulting from a transient increase in bone formation and a sustained decrease in bone resorption (9). Following the rapid accumulation of calcium and phosphate by the skeleton, several observational studies have consistently demonstrated an increase in BMD after PTX (42). The increase in BMD at the tendon attachment site may promote tendon-bone healing leading to improved biomechanical properties. In our patients, it was observed that after PTX, osteoporosis was gradually reversed and BMD began to increase. We assume that after PTX, a massive flow of ions to demineralized bones replenished the calcium deficit and normalized bone turnover which may promote tendon-bone healing. We recommend that once the uremia patient was diagnosed with SHPT, the surgical treatment of PTX is suggested as early as possible after repairing the ruptured tendon.

The current study had several limitations. Firstly, it was a retrospective case series study and the number of cases was relatively small. We did not observe changes in bone metabolism markers after PTX. Secondly, only one patient underwent BMD and IMS examination after PTX, so the recovery of BMD and quadriceps muscle strength could not be quantitatively assessed. Thirdly, all uremia patients with QTR were combined with SHPT and received PTX after QT repair. Further studies are needed to determine the optimal timing of PTX. Finally, we failed to collect the data of uremia patients with SHPT but did not perform PTX which made it impossible to obtain direct evidence of PTX in promoting tendon-bone healing after repair of QT in patients with uremia and SHPT.

## Conclusions

Figure-of-eight trans-osseous sutures with an overlapping tightening suture technique is an economical and effective treatment for spontaneous QTR in patients with uremia and SHPT. PTX may promote tendon-bone healing in patients with uremia and SHPT.

## Data Availability

The datasets presented in this study can be found in online repositories. The names of the repository/repositories and accession number(s) can be found in the article/Supplementary Material.
